# Reconfigurable Optical Signal Processing Based on a Distributed Feedback Semiconductor Optical Amplifier

**DOI:** 10.1038/srep19985

**Published:** 2016-01-27

**Authors:** Ming Li, Ye Deng, Jian Tang, Shuqian Sun, Jianping Yao, José Azaña, Ninghua Zhu

**Affiliations:** 1 Institute of Semiconductors, Chinese Academy of Sciences, No. 35, Tsinghua East Road, Beijing, 100083, China; 2 Microwave Photonics Research Laboratory, School of Information Technology and Engineering, University of Ottawa, 800 King Edward Avenue, ON K1N 6N5, Canada; 3 Institut National de la Recherche Scientifique - Énergie, Matériaux et Télécommunications (INRS-EMT), Varennes, Québec, J3X 1S2 Canada

## Abstract

All-optical signal processing has been considered a solution to overcome the bandwidth and speed limitations imposed by conventional electronic-based systems. Over the last few years, an impressive range of all-optical signal processors have been proposed, but few of them come with reconfigurability, a feature highly needed for practical signal processing applications. Here we propose and experimentally demonstrate an analog optical signal processor based on a phase-shifted distributed feedback semiconductor optical amplifier (DFB-SOA) and an optical filter. The proposed analog optical signal processor can be reconfigured to perform signal processing functions including ordinary differential equation solving and temporal intensity differentiation. The reconfigurability is achieved by controlling the injection currents. Our demonstration provitdes a simple and effective solution for all-optical signal processing and computing.

All-optical circuits for signal processing and computing have attracted great attention in the past years since they could overcome the bandwidth and speed limitations imposed by conventional electronic-based systems^1^,^2^,^3^,^4^,^5^,^6^,^7^,^8^. A promising approach to the implementation of ultrafast all-optical circuit is to emulate the developments in the electronic domain^9^,^10^. Following the electronic component design strategies, many equivalent photonic signal processors, such as temporal differentiators^11^,^12^,^13^,^14^, temporal integrators^15^,^16^,^17^,^18^, Hilbert transformers^19^,^20^,^21^ and ordinary differential equation (ODE) solvers^22^,^23^,^24^,^25^,^26^,^27^,^28^, have been proposed and demonstrated. Two very relevant examples of these fundamental devices are temporal differentiators and ODE solvers. Temporal differentiators can be used to perform real-time differentiation of an optical signal in the optical domain and have been applied to ultrafast signal generation and pulse characterizations^29^,^30^. ODE solvers play an irreplaceable role in virtually any field of science or engineering, such as automatic control and temperature diffusion processes^31^.

Meanwhile, active optical filtering is widely applied in wavelength multiplexed communication and switching systems since it combines the functions of optical amplifiers and channel filters. An active optical filter can be realized using a semiconductor laser with a bias current slightly below its lasing threshold. When a distributed feedback (DFB) semiconductor laser is driven below its lasing threshold, it behaves as a DFB semiconductor optical amplifier (DFB-SOA)^32^. For signal processing, some approaches based on a DFB-SOA have been proposed in the past few years. An optical demultiplexer employing a DFB-SOA with a spectrum selectivity of 9 GHz and an extinction ratio greater than 11 dB was reported^33^. Moreover, a single passband microwave photonic filter (MPF) based on a DFB-SOA was also demonstrated. By adjusting the driven current, a wide tunable center frequency varying from 11–41 GHz was achieved^34^. Very recently, an optical temporal integrator using a DFB-SOA was investigated by simulations. The influences of the system parameters on its energy transmittance and integration error were analyzed^35^. However, those processors are usually designed to perform a specific function without reconfigurability, which represent a fundamental hurdle for the applications in practical signal-processing engines. To tackle this bottleneck, a programmable photonic signal processor chip for radio frequency (RF) application was proposed^36^. By using a grid of tunable Mach-Zehnder couplers interconnected in a two-dimensional mesh network topology, RF filtering with programmable characters can be achieved.

In this paper, we propose and experimentally demonstrate an analog optical signal processor based on a phase-shifted DFB-SOA, to perform two functions including ODE solving and temporal intensity differentiation. When the phase-shifted DFB-SOA is operating below lasing threshold, it could act as a first-order ODE solver with a tunable constant coefficient by tuning the injection current. While the phase-shifted DFB-SOA is operating above the threshold and is operating jointly with an optical filter, it could behave as a temporal intensity differentiator. The proposed signal processor is experimentally evaluated. The experimental results show that the output waveforms are in a good agreement with those from an ideal ODE solver and a temporal differentiator. In addition, the processing errors of our proposed optical signal processor are also analyzed. To the best of our knowledge, it is the first time that a signal processor that is reconfigurable to operate as a temporal intensity differentiator and an ODE solver based on a phase-shifted DFB-SOA.

The schematic diagram of the phase-shifted DFB-SOA is shown in Fig. 1(a). As can be seen, two concatenated identical uniform Bragg gratings with a central 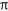-phase-shift between them are built on top of the active layer of the DFB-SOA. Both the front and rear facets of the phase-shifted DFB-SOA are coated with anti-reflection (AR) films and thus the input optical signal can pass through the device. An external current is injected into the phase-shifted DFB-SOA to adjust the gain of the active layer. The corresponding physical image of the packaged phase-shifted DFB-SOA is shown in Fig. 1(b). A temperature control module is fabricated under the DFB-SOA to assure a stable operation of the device.

## Photonic ODE solver

**Figure 2:**
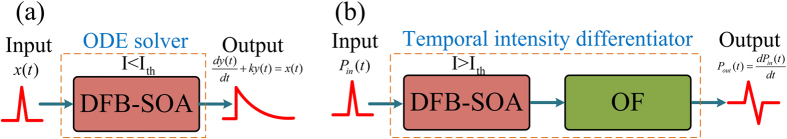
Schematic diagram of the analog optical signal processor. (**a**) The proposed analog optical signal processor is configured as a photonic ODE solver. (**b**) The proposed analog optical signal processor is configured as a photonic temporal intensity differentiator.

First, the proposed phase-shifted DFB-SOA based analog optical signal processor is configured as a photonic ODE solver, as shown in Fig. 2(a). The injection current is below the lasing threshold. An input optical signal with a central wavelength identical to the resonant wavelength of the phase-shifted DFB-SOA is launched into the DFB-SOA through the front facet and then outputted from the DFB-SOA through the rear facet. By adjusting the injection current, the constant coefficient of the ODE can be tuned.

Mathematically, a first-order linear ODE can be expressed as


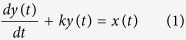


where 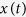 is the input signal, 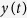 is the output signal, and 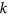 is the constant coefficient. The transfer function of the first-order linear ODE can be numerically obtained by applying Fourier transform to Eq. (1),


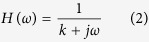


where 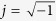 and 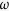 is the optical angular frequency. Therefore, an optical device with a transfer function given by Eq. (2) can be used to solve the first-order linear ODE. According to the transfer matrix method and the coupled-mode theory, the transfer function of a Bragg grating with a 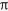 phase-shift at the center of the grating is given by^35^


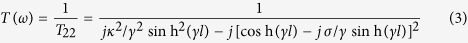


where 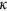 is the coupling coefficient, 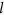 is the length of the Bragg grating and 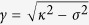. 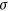 denotes the complex detuning which is given by


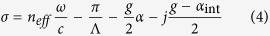


where 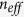 is the effective refractive index, 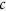 is the velocity of light in vacuum, 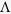 is the grating period, 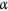 is the linewidth enhancement factor of the active layer, 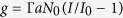 is the power gain coefficient in unsaturated condition, 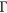 is the confinement factor, 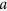 is the differential gain, 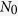 is the carrier density at transmission, 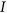 is the injection current of the phase-shifted DFB-SOA, 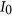 is the current required to reach transparency, and 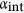 is the internal loss coefficient. When the angular frequency of the input optical signal is very close to the resonant frequency 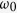 of the phase-shifted DFB-SOA, the transfer function given by Eq. (3) can be rewritten as


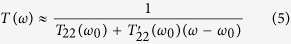


It can be seen from Eq. (5) that the transfer function of the phase-shifted DFB-SOA has an identical form as that in Eq. (2). Therefore, the phase-shifted DFB-SOA can be used to implement a photonic ODE solver. Since the constant coefficient 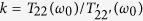 is related to the power gain coefficient 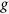 of the active layer, which is dependent on the injection current 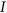, thus the proposed signal processor can solve a first-order linear ODE with a tunable constant coefficient by simply controlling the injection current.

## Photonic temporal intensity differentiator

Then, the proposed phase-shifted DFB-SOA based analog optical signal processor is configured as a photonic temporal intensity differentiator, as shown in Fig. 2(b). The phase-shifted DFB-SOA is biased to operate at the lasing mode, and it is connected with an optical filter (OF). The fundamental principle to achieve temporal intensity differentiation is to use the cross-phase modulation (XPM) in the phase-shifted DFB-SOA to generate a phase-modulated signal and then use the optical filter to perform frequency discrimination.

The phase changes of the lasing wavelength 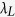 can be written as the sum of phase shifts introduced by carrier density modulation and carrier heating (CH) effects, given by^37^


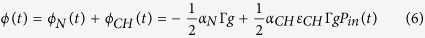


where 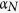, 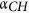 and 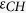 are material-related constants. The frequency deviation 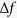 imparted on 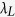 can be expressed as the first order temporal differentiation of the phase 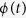



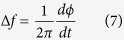


When an OF with a linear power transmission spectrum around 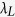 is connected with the phase-shifted DFB-SOA, the frequency deviation of 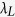 will be converted into intensity modulation at the output of the OF. Assuming that the transfer function of the OF is 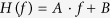, the output optical intensity can be described as


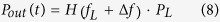


where 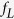 and 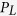 are the frequency and optical power at 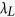, respectively. When the cross-gain modulation (XGM) effect is weak, the parameters 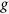 and 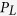 can be considered constants. Accordingly, combining Eqs. (6), (7) and (8), the optical intensity at the output of the OF can be expressed as


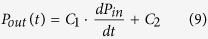


where 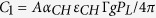, and 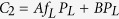. It can be clearly seen from Eq. (9) that a first-order temporal derivative of the input optical signal 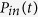 is obtained at the output of the OF. Therefore, the phase-shifted DFB-SOA cascaded with an OF can perform first-order temporal differentiation of the optical intensity of an input optical signal.

## Results

### Photonic ODE solver

**Figure 3:**
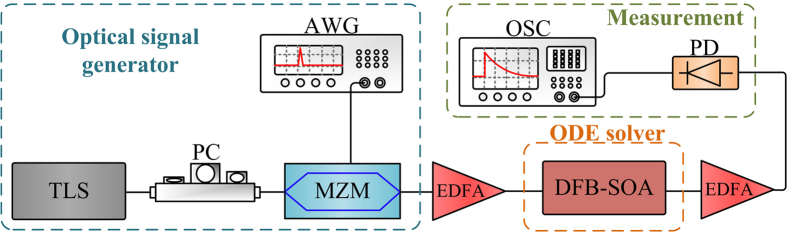
Experimental setup for the photonic ODE solver. TLS: tunable laser source. PC: polarization controller. MZM: Mach-Zehnder modulator. AWG: arbitrary waveform generator. EDFA: erbium-doped optical fiber amplifier. PD: photodetector. OSC: oscilloscope.

We firstly test the operation of the proposed phase-shifted DFB-SOA-based analog optical signal processor reconfigured as a photonic ODE solver. The experimental setup is shown in Fig. 3. It consists of a tunable laser source (TLS), a polarization controller (PC), a Mach-Zehnder modulator (MZM), an arbitrary waveform generator (AWG), a phase-shifted DFB-SOA, two erbium-doped optical fiber amplifiers (EDFAs), a photodetector (PD) and an oscilloscope (OSC). A continuous-wave (CW) light from the TLS with central wavelength tuned to match the resonant wavelength of the phase-shifted DFB-SOA is sent to the MZM via the PC. The polarization state of the light wave is adjusted by the PC to minimize the polarization-dependent loss. The CW light is then sent to the MZM which is driven by an electrical pulse from the AWG. At the output of the MZM, the modulated optical signal is directed to the phase-shifted DFB-SOA. A tunable DC source is employed to generate a tunable current to inject into the phase-shifted DFB-SOA. The EDFAs are utilized to amplify the optical signal to compensate for the insertion losses of the MZM and the phase-shifted DFB-SOA. The optical signal at the output of the phase-shifted DFB-SOA is detected at the PD and monitored by the OSC.

**Figure 4:**
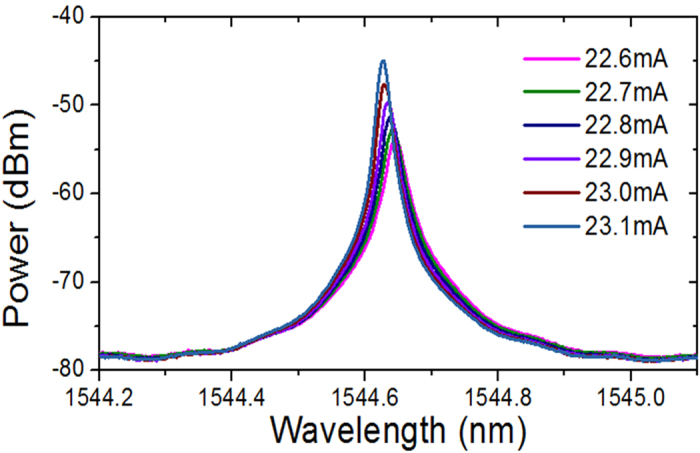
Measured transmission spectra of the DFB-SOA with different injection currents. The injection current to the phase-shifted DFB-SOA ranges from 22.6–23.1 mA. The threshold current is measured to be 23.1 mA.

The measured transmission spectra with different injection currents are shown in Fig. 4. The threshold current of the phase-shifted DFB-SOA is about 23.1 mA with a Q-factor calculated to be about 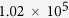. The large insertion loss is mainly resulted from the coupling loss in packaging and propagation loss in active region. It can be seen that as the injection current increases, the transmission spectrum becomes narrow and sharp, resulting the increase of the Q-factor, and the resonant wavelength also shifts toward a shorter wavelength side. This is due to the refractive index change in the active layer due to carrier injection. When the injection current to the phase-shifted DFB-SOA is 22.6 mA, the 3-dB bandwidth is about 0.026 nm with a resonant wavelength of 1544.646 nm. When the injection current is changed to be 23.1 mA, the 3-dB bandwidth becomes 0.015 nm with a resonant wavelength of 1544.628 nm. The change in the transmission spectra under various injection currents makes the first-order linear ODE solver to operate with a tunable constant coefficient. Moreover, the operating bandwidth of the ODE solver is determined by the frequency difference between the two minimum points of the transmission spectrum, which is measured to be around 95.75 GHz.

**Figure 5:**
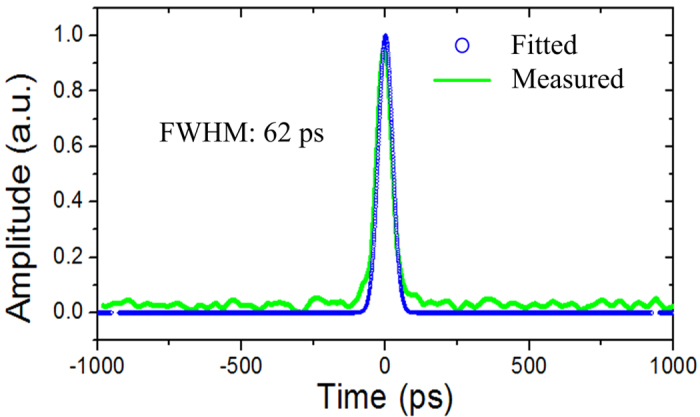
Input waveforms for the photonic ODE solver. A Gaussian pulse with a FWHM of 62 ps is used as the input optical signal for the photonic ODE solver. Blue dotted line: fitted input waveform. Green solid line: measured input waveform.

**Figure 6:**
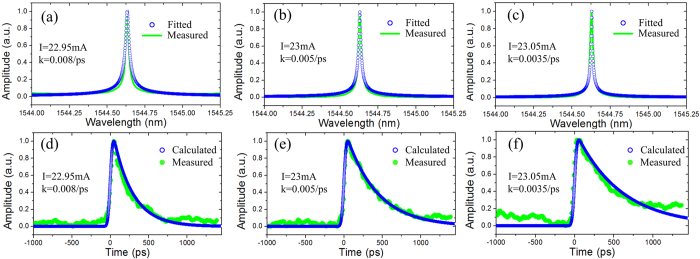
Experimental results for the photonic ODE solver. The transmission spectra of the phase-shifted DFB-SOA with different injection currents of (**a**) 22.95 mA, (**b**) 23 mA, and (**c**) 23.05 mA. Blue dotted line: fitted transmission spectrum. Green solid line: measured transmission spectrum. The corresponding output waveforms for the injection currents of (**d**) 22.95 mA, (**e**) 23 mA, and (**f**) 23.05 mA. Blue dotted line: calculated output waveforms. Green solid line: measured output waveforms.

In our experiment, Gaussian pulse trains with a period of 5 ns are used as the input optical signal. The measured and fitted input waveforms are shown in Fig. 5. It can be seen that the full width at half maximum (FWHM) of the Gaussian pulse is measured to be 62 ps. When the injection current to the phase-shifted DFB-SOA is 22.95 mA, we get the measured and fitted transmission spectra, which are shown in Fig. 6(a). It is worth noting that the corresponding constant coefficient 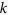 of 0.008/ps is calculated from the fitted transmission spectrum based on the ideal transfer function of the first-order linear ODE, i.e., Eq. (2). The calculated output waveform is depicted in Fig. 6(d) for comparison with the measured output waveform. One can clearly see that a good agreement between the calculated and the measured output waveforms is reached. When the injection current is changed to 23 and 23.05 mA, the transmission spectrums are shifted to a longer wavelength, as illustrated in Fig. 6(b,c). The corresponding constant coefficients are calculated to be 0.005/ps and 0.0035/ps, respectively. The calculated and measured output waveforms are also shown in Fig. 6(e,f), respectively. In addition, the measured output waveforms are broadened with the decreased constant coefficients, which are resulted from the increased gain coefficients due to the increased injection currents. For different values of the constant coefficient, it can be obviously seen that all the measured output waveforms fit well with the calculated results. Therefore, the capability to solve first-order linear ODE with tunable constant coefficient by using a single phase-shifted DFB-SOA is verified.

### Photonic temporal intensity differentiator

**Figure 7:**
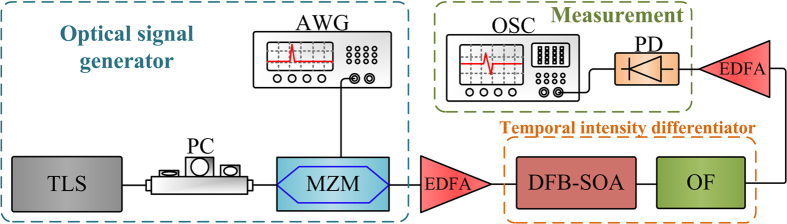
Experimental setup for the photonic temporal intensity differentiator. TLS: tunable laser source. PC: polarization controller. MZM: Mach-Zehnder modulator. AWG: arbitrary waveform generator. OF: optical filter. EDFA: erbium-doped optical fiber amplifier. PD: photodetector. OSC: oscilloscope.

**Figure 8:**
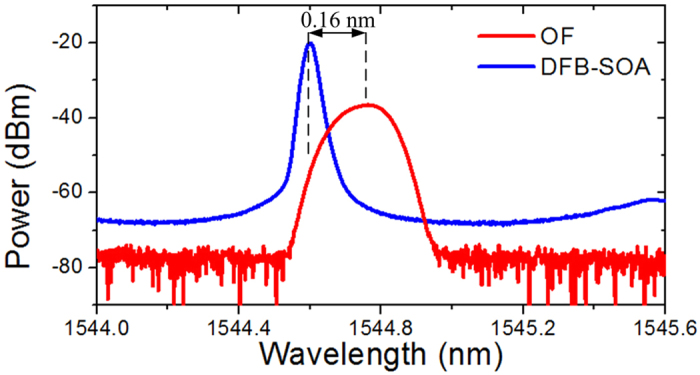
Spectra of the phase-shifted DFB-SOA and the OF. Blue line: Measured spectrum of the phase-shifted DFB-SOA with injection current of 40 mA. Red line: measured transmission spectrum of the OF.

We also test the operation of the proposed phase-shifted DFB-SOA-based analog optical signal processor reconfigured as a temporal intensity differentiator with the experimental setup, as shown in Fig. 7. An optical signal with a central wavelength of 1544 nm is sent into the temporal intensity differentiator. The phase-shifted DFB-SOA is driven by an injection current at 40 mA and thus is operating at the lasing mode. The lasing wavelength is measured to be 1544.6 nm, as shown in Fig. 8. An OF is cascaded with the phase-shifted DFB-SOA as a frequency discriminator to extract the chirp variation of the phase-modulated light wave from the phase-shifted DFB-SOA. As can be seen from Fig. 8, the wavelength offset between the center of the OF and phase-shifted DFB-SOA is about 0.16 nm, showing that the lasing wavelength of the phase-shifted DFB-SOA is located at the positive slope of the spectrum of the OF. Finally, the differentiated optical signal is detected at the PD and monitored by the OSC.

**Figure 9:**
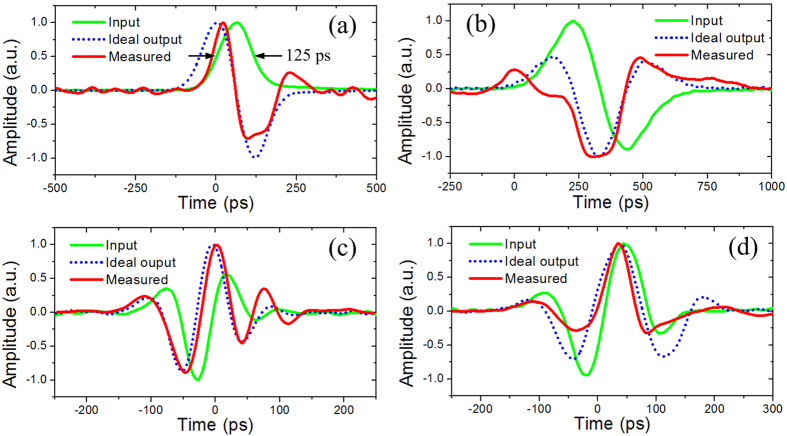
Experimental results for the temporal intensity differentiator. (**a–d**) are output waveforms for different input optical signals. Green solid line: input waveforms. Blue dotted line: ideal output waveforms. Red solid line: measured output waveforms.

Figure 9 shows the measured pulses at the output of the proposed temporal intensity differentiator for different input signals. When a Gaussian-like pulse with a FWHM of 125 ps is launched into the temporal intensity differentiator, as shown in Fig. 9(a), a monocycle-like pulse is obtained at the output. For comparison, an ideal output pulse (derivative of the input pulse by simulation) is also depicted in Fig. 9(a). It can be seen that a good agreement between the experimentally generated pulse and the ideal output pulse is reached. The root mean square error (RMSE) is calculated, which is about 16.4%. The other experimentally generated pulses for different input signals are shown in Fig. 9(b–d). The RMSEs are calculated to be 14.2%, 5.4% and 10.92%, respectively. Note that the output pulses are the temporal derivation of the optical intensity of the input pulses, not the optical field, thus the proposed phase-shifted DFB-SOA-based analog optical signal processor can be configured to operate as a first-order temporal intensity differentiator.

## Discussion

**Figure 10:**
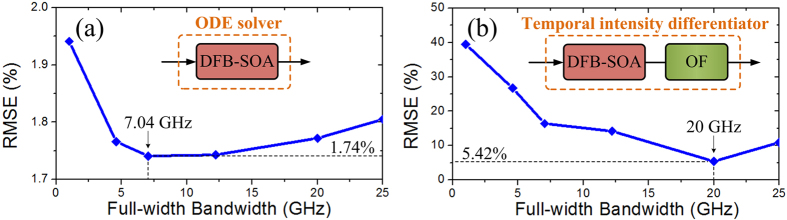
Device processing error. (**a**) Calculated RMSEs for the photonic ODE solver with different bandwidth of the input signals. (**b**) Calculated RMSEs for the photonic temporal intensity differentiator with different bandwidth of the input signals.

The operation bandwidth of the proposed analog optical signal processor based on a phase-shifted DFB-SOA is evaluated. It is done by calculating the processing errors as a function of the full-width bandwidth of the input optical pulses. When the processor is configured as a first-order ODE solver under an injection current of 23.05 mA, the RMSEs are calculated, which are shown in Fig. 10(a). Note that in the calculations, we assume an ideal Gaussian input pulse and the RMSEs are calculated as the difference between the temporal intensities of the simulated output pulse based on the measured spectrum transfer functions and the ideal output waveform. As can be seen, the photonic ODE solver can provide optimal operations with minimum error of 1.74% with a full-width bandwidth of 7.04 GHz. The processing error is mainly resulted form the spontaneous emission of the phase-shifted DFB-SOA. The photonic ODE solver can work beyond the optimal processing bandwidth at the cost of an increased RMSE. When the processor is configured as a temporal intensity differentiator, the calculated RMSEs are depicted in Fig. 10(b). One can see that, the minimum operation error is calculated to be 5.42% with a full-width bandwidth of 20 GHz. The errors are mainly caused by the cross-gain modulation (XGM) effect^38^ and the nonlinear slope of the OF. By operating the phase-shifted DFB-SOA in small XGM region and employing an OF with linear power transmission spectrum, the processing error of the temporal intensity differentiator can be largely reduced. In order to assure operation stability of the proposed processor, the stable current control unit and temperature control unit are needed. It is worth noting that, since the proposed photonic first-order ODE solver based on a phase-shifted DFB-SOA is a linear device, a high-order ODE solver with tunable constant coefficient can be achieved by simply cascading multiple phase-shifted DFB-SOAs. In addition, if the phase-shifted DFB-SOA is driven very close to the lasing threshold current, it can act as an optical temporal integrator. Moreover, besides the intrinsic interest in future ultrafast optical computing and information processing systems, the proposed photonic temporal intensity differentiator also has the potential for wireless application in microwave photonic processing for the generation of ultrawideband (UWB) pulses such as UWB monocycle and doublet pulses^39^.

In summary, we have proposed and experimentally demonstrated an analog optical signal processor based on a phase-shifted DFB-SOA, to perform functions of ODE solving and temporal intensity differentiation. A first-order ODE solver with tunable constant coefficients was achieved. By tuning the injection current to the phase-shifted DFB-SOA, the constant coefficient of the first-order ODE is tuned from 0.0035/ps–0.008/ps. A temporal intensity differentiator was also realized by cascading an OF with the phase-shifted DFB-SOA that is operating in the lasing mode. A good agreement between the experimental results and the calculated output waveforms was obtained. In addition, the processing error as a function of bandwidth of the input optical signal was also analyzed. Furthermore, thanks to the capacity of integration with other optical components on InP/InGaAsP platform, the proposed optical signal processor has a high potential to provide simple and effective solutions for all-optical signal processing and computing.

## Methods

### Input waveform

We used a TLS (Agilent 8164B) with linewidth less than 100 kHz as the light source. The tuning resolution of the TLS is 0.01 nm, which enables us to precisely align the signal wavelength to the resonant wavelength of the phase-shifted DFB-SOA. The CW light is then sent to the MZM which is driven by an electrical pulse from the AWG (Tektronix 70001A) with sampling rate of 50 GS/s. The DC bias of the MZM is adjusted to below the quadrature point and the amplitude of electrical signal is less than the half-wave voltage of the MZM which is measured to be 6 V. In this way, an arbitrary optical signal can be generated at the output of the MZM by programming the AWG.

## Additional Information

**How to cite this article**: Li, M. *et al.* Reconfigurable Optical Signal Processing Based on a Distributed Feedback Semiconductor Optical Amplifier. *Sci. Rep.* **6**, 19985; doi: 10.1038/srep19985 (2016).

## Figures and Tables

**Figure 1:**
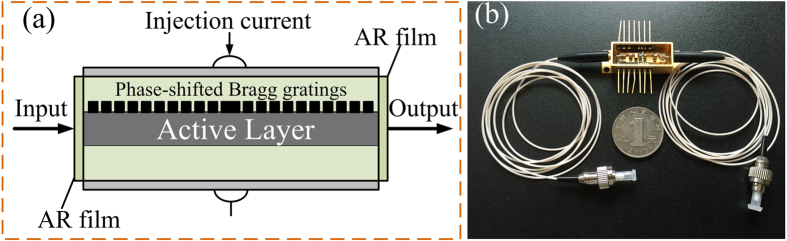
The phase-shifted DFB-SOA. (**a**) Schematic diagram and (**b**) the physical image of the packaged phase-shifted DFB-SOA.
